# Established fibrous peritoneal metastasis in an immunocompetent mouse model similar to clinical immune microenvironment of gastric cancer

**DOI:** 10.1186/s12885-020-07477-x

**Published:** 2020-10-20

**Authors:** Daisuke Fujimori, Jun Kinoshita, Takahisa Yamaguchi, Yusuke Nakamura, Katsuya Gunjigake, Takashi Ohama, Koichi Sato, Masami Yamamoto, Tetsuya Tsukamoto, Sachiyo Nomura, Tetsuo Ohta, Sachio Fushida

**Affiliations:** 1 Department of Gastroenterological Surgery, Graduate School of Medical Science, Kanazawa University, 13-1 Takara-machi, Kanazawa, 920-8641 Japan; 2 Laboratory of Veterinary Pharmacology, Joint Faculty of Veterinary Medicine, Yamaguchi University, Yamaguchi, Japan; 3 Department of Applied Science, School of Veterinary Nursing and Technology, Nippon Veterinary and Life Science University, Musashino, Japan; 4 Department of Diagnostic Pathology, Fujita Health University School of Medicine, Toyoake, Japan; 5 Department of Gastrointestinal Surgery, Graduate School of Medicine, The University of Tokyo, Tokyo, Japan

**Keywords:** Gastric cancer, Peritoneal metastasis, Immunocompetent mouse model, Tumor microenvironment

## Abstract

**Background:**

Peritoneal metastasis (PM) in gastric cancer (GC) is characterized by diffusely infiltrating and proliferating cancer cells accompanied by extensive stromal fibrosis in the peritoneal space. The prognosis of GC with PM is still poor regardless of the various current treatments. In order to elucidate the cause of difficulties in PM treatment, we compared the tumor immune microenvironment (TME) in primary and PM lesions in GC. In addition, a PM model with fibrous stroma was constructed using immunocompetent mice to determine whether its TME was similar to that in patients.

**Methods:**

Immuno-histochemical analyses of infiltrating immune cells were performed in paired primary and PM lesions from 28 patients with GC. A C57BL/6 J mouse model with PM was established using the mouse GC cell line YTN16 either with or without co-inoculation of mouse myofibroblast cell line LmcMF with α-SMA expression. The resected PM from each mouse model was analyzed the immunocompetent cells using immunohistochemistry.

**Results:**

The number of CD8^+^ cells was significantly lower in PM lesions than in primary lesions (*P* < 0.01). Conversely, the number of CD163^+^ cells (M2 macrophages) was significantly higher in PM lesions than in primary lesions (*P* = 0.016). Azan staining revealed that YTN16 and LmcMF co-inoculated tumors were more fibrous than tumor with YTN16 alone (*P* < 0.05). Co-inoculated fibrous tumor also showed an invasive growth pattern and higher progression than tumor with YTN16 alone (*P* = 0.045). Additionally, YTN16 and LmcMF co-inoculated tumors showed lower infiltration of CD8^+^ cells and higher infiltration of M2 macrophages than tumors with YTN16 alone (*P* < 0.05, *P* < 0.05). These results indicate that LmcMF plays as cancer-associated fibroblasts (CAFs) by crosstalk with YTN16 and CAFs contribute tumor progression, invasion, fibrosis, and immune suppression.

**Conclusions:**

This model is the first immunocompetent mouse model similar to TME of human clinical PM with fibrosis. By using this model, new treatment strategies for PM, such as anti-CAFs therapies, may be developed.

## Background

Gastric cancer (GC) is one of the most common malignancies worldwide, with an estimated 1,033,701 new cases and 782,685 associated deaths in 2018 according to the International Agency for Research on Cancer [1]. Peritoneal metastasis (PM) is the most common mode of metastasis in gastric cancer and a critical indicator of poor prognosis [2–4]. Although various approaches to the treatment of PM have been assessed, including systemic and/or intraperitoneal chemotherapy [5–8], hyperthermic intraperitoneal chemotherapy (HIPEC) [9, 10], and aggressive surgery (peritonectomy) [11, 12], satisfactory outcomes have not been achieved. PM is characterized by rapid infiltration and proliferation of cancer cells accompanied by extensive stromal fibrosis, causing potentially fatal disorders such as bowel obstruction, hydronephrosis, and jaundice [13]. Fibrosis interferes with drug delivery due to high intratumoral pressure [14]. These fibrous stroma are conducted by crosstalk between cancer cells and cancer-associated fibroblasts (CAFs) [15–17]. CAFs also induce endothelial mesenchymal transduction (EMT) in cancer cells, result to produce the invasion ability and chemo-resistance in cancer cells. Besides, cytokines and chemokines derived from CAFs interact tumor immune microenvironment (TME), which affect not only chemo-sensitivities but also efficacy of immune checkpoint inhibitors (ICIs).

Recently, ICIs have become a major tools of cancer therapy tool. ICI therapies have been shown to be extremely effective against numerous malignancies, such as melanoma, renal cell carcinoma, non-small cell lung carcinoma (NSCLC), and Hodgkin disease [18–21]. The anti-programmed death-1 (PD-1) antibody, nivolumab, showed an 11% of response rate and 40% of disease control rate for late line of metastatic GC patients in the ATTRACTION2 trial [22]. Generally, the efficacy of anti-PD-1 antibody is believed to depend on the tumor microenvironment with PD-ligand 1 (PD-L1) expression in cancer cells and the presence of tumor-infiltrating lymphocytes (TILs), mediating adaptive immune resistance [23]. This tumor immune microenvironment (TME) phenotype was found in 10–11% of Japanese GC patients [24, 25], which is consistent with data from the ATTRACTION2 trial [22]. However, the antitumor response to nivolumab was independent of tumor PD-L1 status in this trial. These results suggest that other components of TME, such as cancer-associated fibroblasts (CAFs) and tumor-associated macrophages (TAMs), might affect the immune system in PM. To obtain a favorable outcome using ICIs, tailoring combined therapy with modification of the TME will need to be considered. In the present study, we compared the TME in primary and peritoneal lesions to elucidate the cause of difficulties in treatment of metastatic GC. Additionally, a PM model with fibrous stroma features similar to those of human clinical samples was established using immunocompetent mice. The model was used to confirm whether the TME was similar to clinical PM for the development of new treatment strategies.

## Methods

### Patients and resource of samples

We performed immunostaining of paired primary lesions and peritoneal lesions from 28 GC patients with PM at our hospital from 2009 to 2016, and compared their immune environments. Tissue samples from primary lesions were obtained from biopsy specimens during upper gastrointestinal endoscopy, and peritoneal lesion were obtained from sampling during staging laparoscopy. All specimens were obtained before chemotherapy. Prior to this research, written informed consent was obtained from each patient. This study was approved by the Institutional Review Board of Kanazawa University Graduate School of Medical Science (study permission number 2789).

### Cell lines and cell culture

YTN16 is a gastric cancer cell line transplantable into C57BL/6 mice. YTN16 cells were established from subcutaneous tumors by injection of primary cultured cells derived from a mouse gastric adenocarcinoma. Mouse gastric tumors were established in p53 heterozygous knockout C56BL/6 mice by addition of *N*-Methyl-*N*-nitrosourea (MNU) to the animals’ drinking water [26]. The resulting tumor cells were cultured in high-glucose Dulbecco’s modified Eagle medium (DMEM, Sigma-Aldrich Japan, Tokyo, Japan) containing 1.0 mL/L MITO (Coning Japan, Tokyo), 10 mL/L L-Glutamine, 10 mL/L Penicillin/Streptomycin and 10% fetal bovine serum (FBS), on plastic dishes coated with Type I collagen solution (0.5% Atelocollagen Acidic Solution IPC-50; Koken Co., Ltd., Japan) at 37 °C in 5% CO_2_ atmosphere.

Mouse intestinal myofibroblast cell lines (LmcMFs) derived from mouse colonic mucosa were established by Takashi Ohama and Koichi Sato, Laboratory of Veterinary Pharmacology, Joint Faculty of Veterinary Medicine, Yamaguchi University [27]. Cells were cultured in DMEM containing 10% FBS at 37 °C in 5% CO_2_ atmosphere.

### Co-culture

Indirect co-cultures were established as follows. YTN16 cells were seeded on a 6-well plate, while LmcMF cells were seeded in 1-μm pore-size Boyden chambers (BD Falcon, Franklin Lakes, NJ), both at a density of 1 × 10^5^ cells per well or chamber in DMEM or high-glucose DMEM containing 10% FBS, respectively. After 24 h, the cells were washed twice with PBS, the chambers were placed into the wells of the plates, and the plates were incubated for 5 days in 2 mL of DMEM or high-glucose DMEM.

### Mouse allograft model

The animal use proposal and experimental protocol (AP-183944) was reviewed and approved by the Animal Care and Use Committee of Kanazawa University. All animal experiments were performed in accordance with the standard guidelines of Kanazawa University. Female C57BL/6 J mice (20 g, 6–8 weeks old) were purchased from Charles River Laboratories, Inc., Yokohama, Japan. The mice were housed with a 12 h day-night cycle in a temperature-(21 °C) and humidity-(50%) controlled room of the animal experimental institute. All mice were kept in individually ventilated cages, fed with sterile standard food and water ad libitum. Mice were randomly distributed into YTN16 inoculated group (*n* = 6) and YTN16 with LmcMF co-inoculated group (*n* = 6). To establishing peritoneal metastatic models, 1 × 10^7^ of YTN16 cells alone in 1 mL of high-glucose DMEM were inoculated intraperitoneally under isoflurane anesthesia on day 0 as YTN16 inoculated group. YTN16 cells were co-cultured with an equivalent number of LmcMFs for 5 days, and a total of 1 × 10^7^ cells in 1 mL of the same medium were then inoculated same manner as YTN16 inoculated group. In this study, total inoculated cell counts were aligned for comparing tumor weights because tumors consisted of both cancer cells and stroma cells including LmcMFs. After inoculation, on day 14, the mice were euthanized with isoflurane and cervical dislocation, and tumors were removed for weight calculation and immunohistochemical examination.

### Immunohistochemistry

Tumor specimens were fixed in 10% neutral buffered formalin and embedded in paraffin. Sections were stained with hematoxylin and eosin (H&E) and Azan stain for assessment of fibrosis, while the expression of various antigens was assessed immunohistochemically. Deparaffinizing sections were pretreated by autoclaving in 10% citric acid buffer at 120 °C for 15 min. Following treatment with protein block serum (Dako Co., Kyoto, Japan) for 10 min and incubation with 2% skim milk for 30 min to block nonspecific reactions, sections were incubated with primary antibody at 4 °C overnight. Information on the antibodies used is listed in Table 1. After the sections were washed in PBS, immunoreactivity was visualized using EnVision reagent (Dako Co.), and the slides were developed with diaminobenzidine and counterstained with hematoxylin. All sections were examined using a fluorescence microscope (Olympus, Tokyo, Japan). We used Olympus PLFLN 100X as objective lenses, DP70-SET as microscope digital camera, and ‘cell Scans Standard’ as acquisition software. The degree of fibrosis was calculated as a percentage of fibrosis within the whole section in all samples using a BZ-9000 BZII microscope (Keyence, Osaka, Japan). We used CFI Plan Apoλ and CFI Plan Flour as objective lenses, BZ-X Filter GFP as filter, 2/3 in. 2.83 million-pixel monochrome CCD as detectors, and ‘BZ-X Viewer’ as acquisition software.

**Table 1:** List of used antibodies

Antibody	Product code	Origin	Dilution	Source
Human CD4	M7310	mouse IgG mab^a^	1:100	Nichirei, Tokyo, Japan
Human CD8	ab4055	rabbit IgG pab^b^	1:200	Abcam, Tokyo, Japan
Human CD163	NCL-L-CD163	mouse IgG mab	1:200	Leica Bio, Tokyo, Japan
Mouse α-SMA	ab5694	rabbit IgG pab	1:1000	Abcam, Tokyo, Japan
Mouse CD4	ab183685	rabbit IgG mab	1:1000	Abcam, Tokyo, Japan
Mouse CD8	ab209775	rabbit IgG mab	1:2000	Abcam, Tokyo, Japan
Mouse CD163	ab182422	rabbit IgG mab	1:500	Abcam, Tokyo, Japan

^a^*mab* Monoclonal antibody, ^b^*pab* Polyclonal antibody

### Quantification of immunostaining parameters

Data were obtained by manually counting positively stained cells in five non-overlapping intratumoral fields. Due to the small size of specimens, clinical specimens were analyzed under × 400 magnification to avoid counting the non-tumoral fields. Stained cells in mouse model tumors were accessed under × 100 magnification for CD4^+^ and CD8^+^ cells, and under × 200 magnification for CD163^+^ cells. All immunostaining was interpreted by two independently (JK and TY).

### Statistical analysis

Statistical analyses were conducted using SPSS statistical software, version 23 (IBM Corp., Armonk, NY, USA). Comparison of peritoneal tumor weight was made using Student’s *t*-test. Differences among the cell count and fibrotic area data sets were evaluated using the Mann-Whitney U-test. *P* values less than 0.05 indicated statistical significance.

## Results

### Patient characteristics

Patient characteristics are shown in Table 2. The median age of the study population was 66 years (range, 27–83 years). Overall, 17 men and 11 women were enrolled in the study. Twenty-five of the twenty-eight patients (89%) had diffuse type GC (Bormann 3 or 4), while the other three patients showed intestinal type GC based on the Lauren classification. According to the Japanese Classification of Gastric Carcinoma 15th edition, the P statuses were P1a in 11 cases, P1b in 4 cases, and P1c in 13 cases.

**Table 2:** Patient characteristics

Characteristic	
Age	
Median	66
Range	27–83
Sex	
Male	17
Female	11
Macroscopic type	
2	3
3	11
4	14
Histological type (Lauren)	
Intestinal	3
Diffuse	25
Degree of peritoneal metastasis^a^	
P1a	11
P1b	4
P1c	13

^a^peritoneal metastasis was classified according to the 15th edition of the General Rules for Gastric Cancer Study of the Japanese Research Society for Gastric Cancer

P1a: greater omentum, lesser omentum, anterior lobe of the transverse colon membrane, or membrane of the pancreatic surface or spleen, P1b: a few scattered metastases to upper abdominal peritoneum, namely, the parietal peritoneum close to the umbilical side and the visceral peritoneum close to the cranial transverse colon, P1c: many metastases to middle or lower peritoneum

### Immunostaining of GC primary and peritoneal lesions

**Fig. 1:**
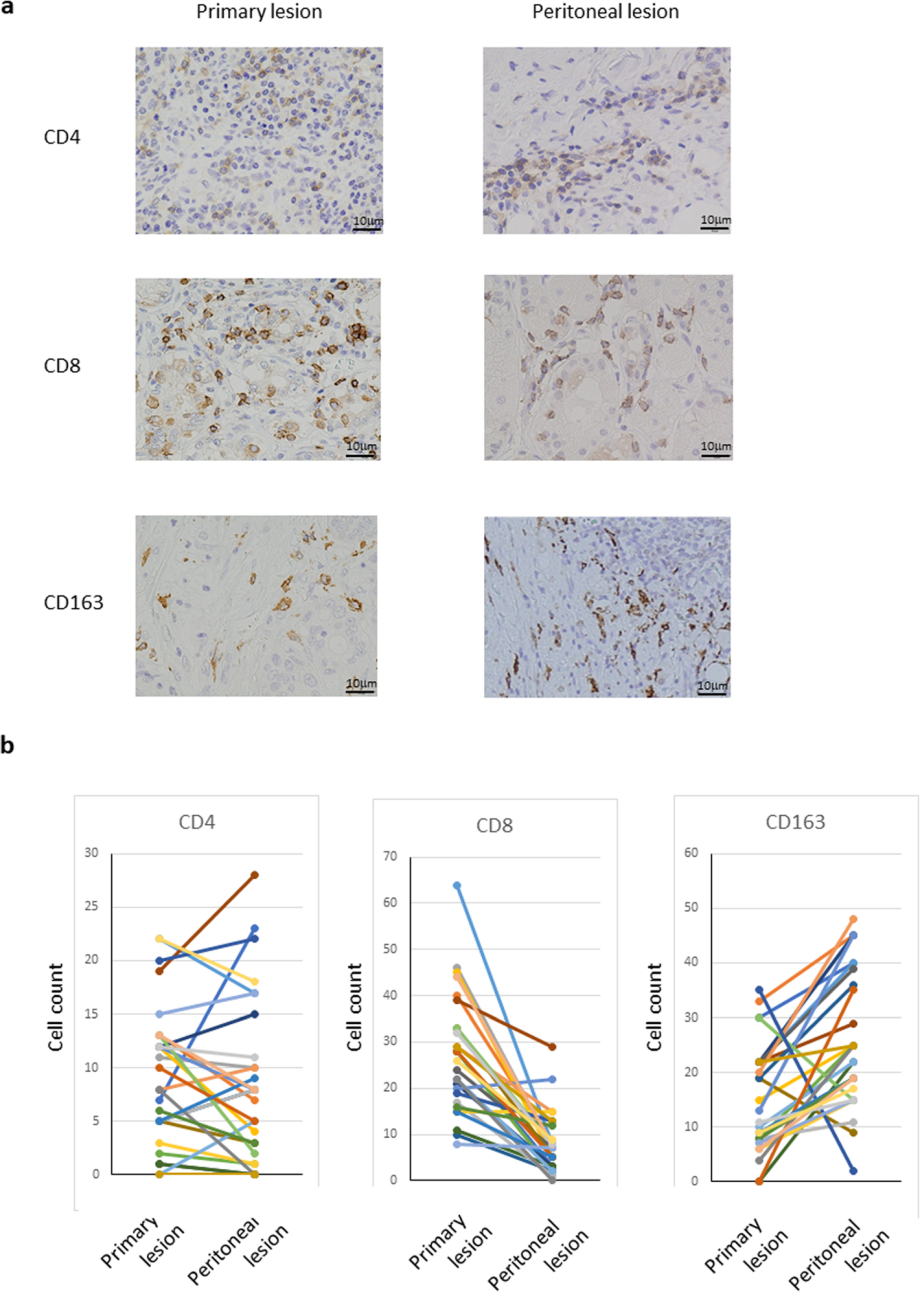
**a** Representative microscopic images of CD4^+^, CD8^+^ and CD163^+^ cells in primary and peritoneal lesions of gastric cancer. Each cell was determined by immunohistochemically (original magnification × 400). **b** Comparisons of tumor immune microenvironment between primary and peritoneal lesions in gastric cancer patients. Each cell was measured and shown as average count of five non-overlapping tumor areas. There were significant differences in CD8^+^ and CD163^+^ cell count between primary and peritoneal lesions (*P* = 0.01, *P* = 0.016).

We performed immunostaining of biopsy specimens of primary and peritoneal lesions to analyze the microenvironment. We used antibodies against CD4 and CD8 as markers for lymphocytes and the CD163 antibody as a marker for M2 macrophages. Representative microscopic views of the peritoneal lesions are shown in Fig. 1a. There was no significant difference in the number of CD4^+^ cells between primary and peritoneal lesions (8.9 ± 5.5 vs. 7.4 ± 7.7). The number of CD8^+^ cells observed in the peritoneal lesions was significantly lower than in the primary lesions (28.3 ± 12.7 vs. 8.7 ± 7.2, *P* = 0.01). CD163 positive cells in the peritoneal lesion were significantly higher than in the primary lesion (18.4 ± 2.5 vs. 32.1 ± 5.0, *P* = 0.016) (Fig. 1b).

### Establishment of fibrous allograft model

**Fig. 2:**
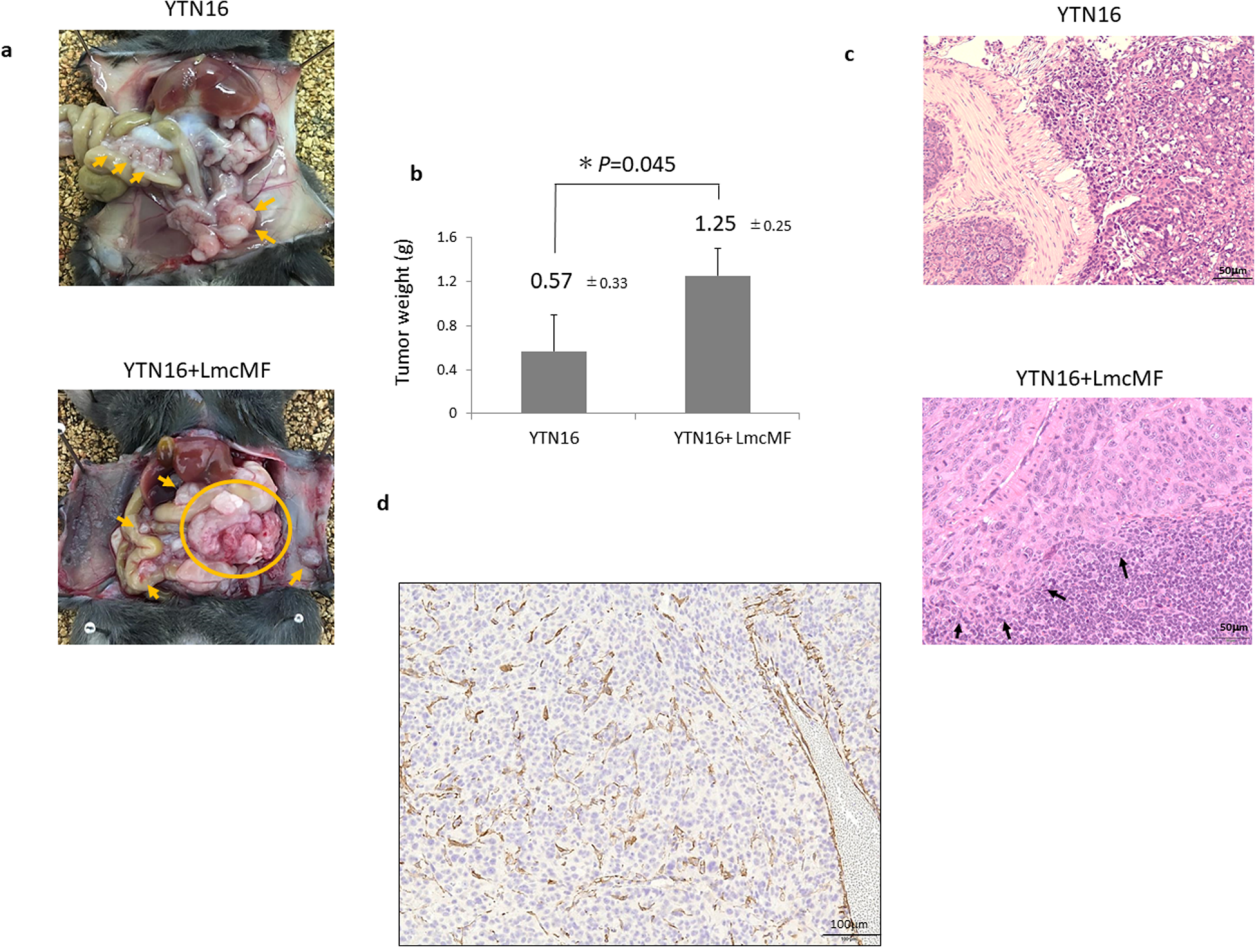
**a** Representative images depicting two pattern of allograft models at day 14. Macroscopic views of peritoneal nodules (*Arrows & inside circle*). **b** Peritoneal tumor volume were compared with the Mann-Whitney U test. Results are expressed as the mean ± SD (*n* = 6). **c** Histological examination of peritoneal allograft models by hematoxylin and eosin (H&E) staining in each tumor (original magnification × 200). Co-inoculated tumor (YTN16 + LmcMF) disappeared splenic capsule resulted to invade the splenic lymph follicle (*Arrows*). **d** α-SMA expressing fibroblasts in co-inoculated tumors (original magnification × 200)

All mice were successfully established peritoneal tumors in each group and were analyzed using their datasets of day 14. YTN16 cells were co-cultured with LmcMF and co-inoculated intraperitoneally into mice. As shown in Fig. 2a and b, the tumor weight of peritoneal tumors from mice co-inoculated with YTN16 and LmcMF was significantly higher than that of tumors from mice inoculated with YTN16 alone (0.57 ± 0.33 g vs. 1.25 ± 0.25 g, *P* = 0.045). Microscopic findings of peritoneal tumors in co-inoculated mice showed invasive progression into spleen, but tumors in mice inoculated with YTN16 alone showed expansive progression, not invasive (Fig. 2c). α-SMA expressing fibroblasts were found in co-inoculated peritoneal tumors as CAFs (Fig. 2d, Additional file 1).

**Fig. 3:**
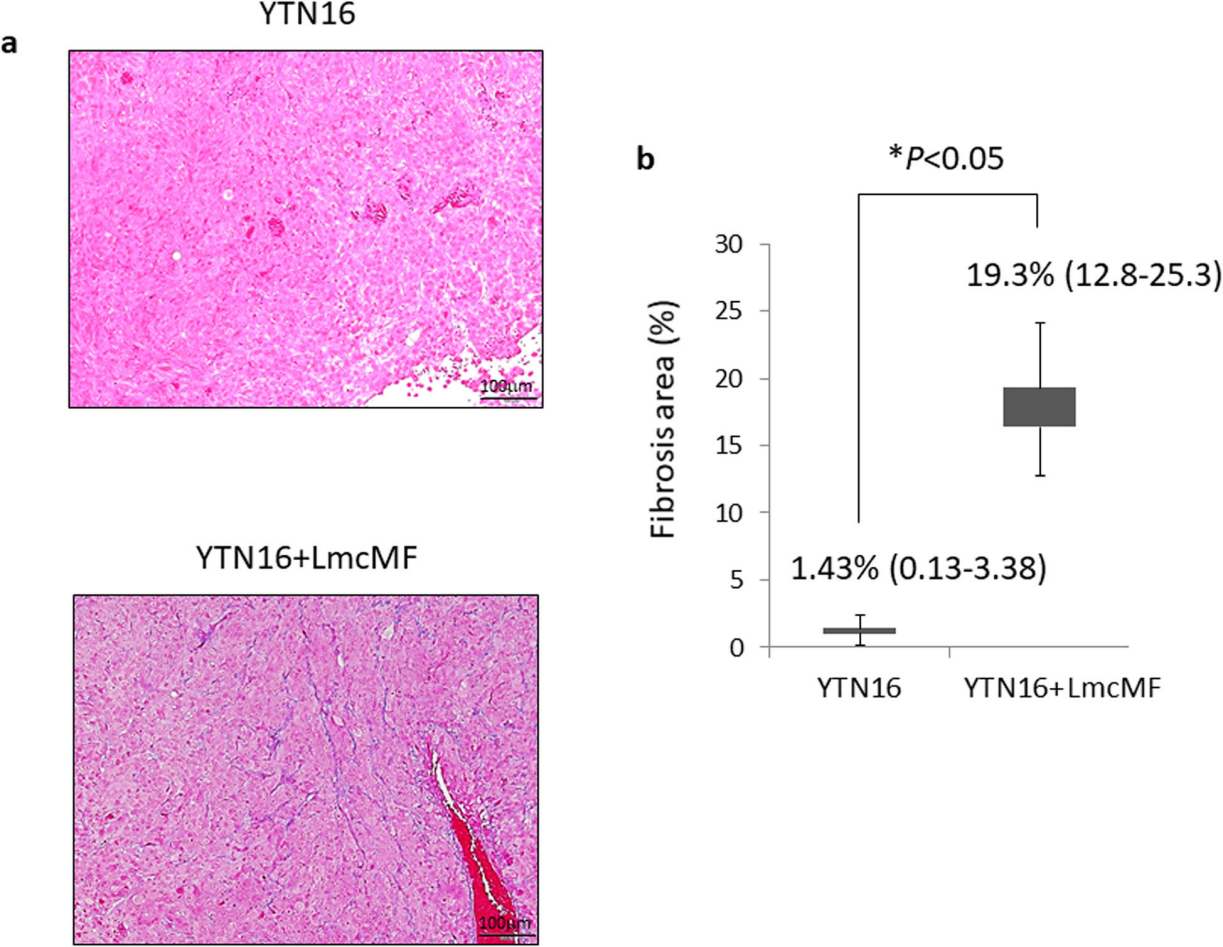
Fibrotic change in each tumors of peritoneal allograft models. **a** Microscopic views of mouse allograft tumors (original magnification × 100). Fibrotic tissues were determined by Azan staining. **b** Fibrotic areas were measured and are shown as a percentage (*fibrotic area/whole section area*). Results are expressed as the mean ± SD (n = 6)

The degree of fibrosis in the tissues was compared by Azan staining (Fig. 3a, Additional file 2). Areas that stained blue were regarded as fibrosis, and the percentage of stained tissue was compared. In the YTN16 control group, some fibrosis was observed, but in the co-inoculated group (YTN16 and LmcMF), the fibrotic area was significantly increased in peritoneal tumors (1.4%; range 0.13–3.38 vs. 19%; range 12.8–25.3, *P* < 0.05) (Fig. 3b). Co-inoculated LmcMF cells that were pretreated with PKH staining were confirmed as a component of fibrous tumors (data not shown).

### Analysis of immune microenvironment of mouse fibrous tumor

**Fig. 4:**
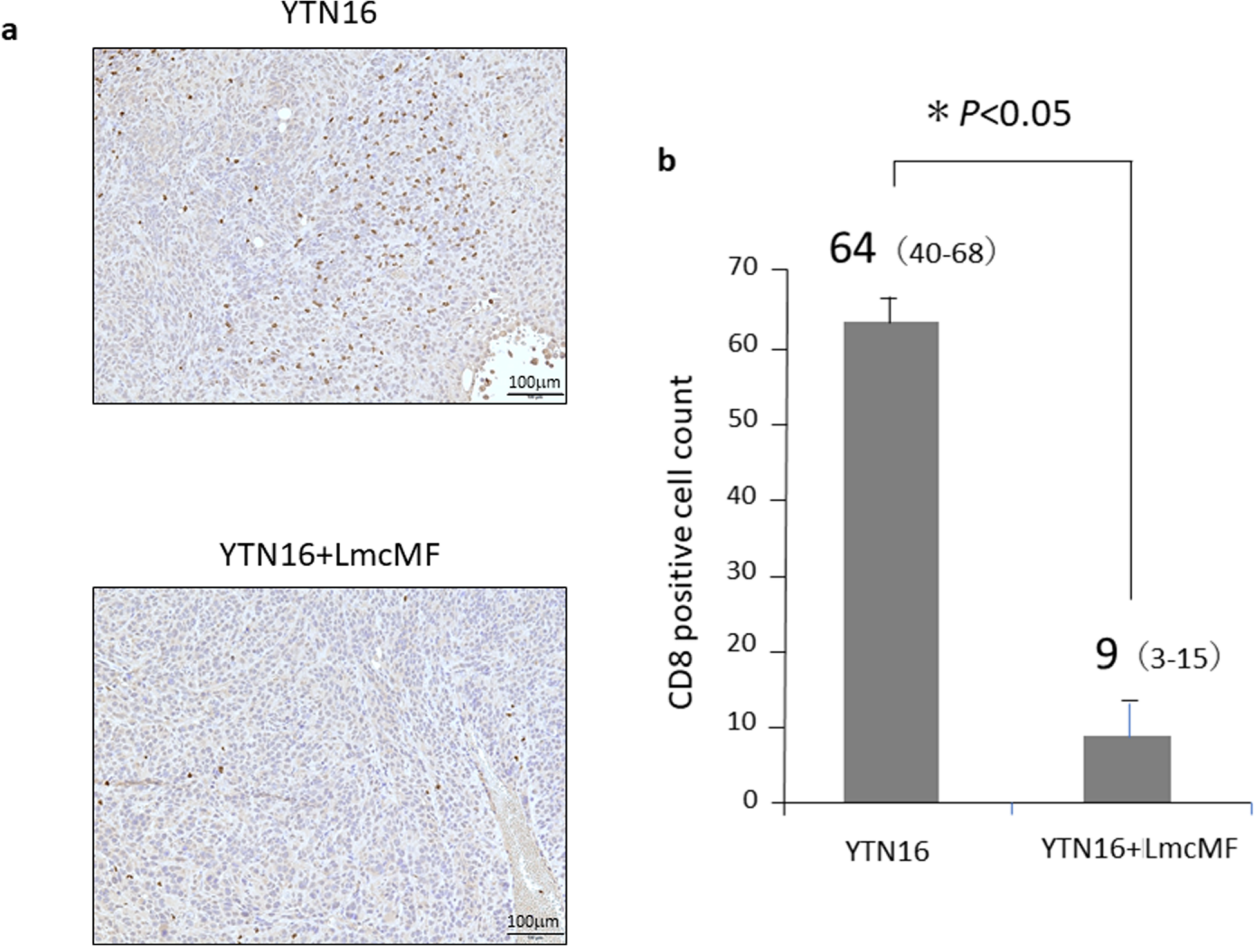
CD8^+^ cell infiltration in each tumors of peritoneal allograft models. **a** Microscopic views of mouse allograft tumors (original magnification × 100). CD8^+^ cells were determined by immunohistochemically. **b** Number of infiltrated CD8^+^ cells were measured and shown as average count of three non-overlapping tumor areas. Results are expressed as the mean ± SD (n = 6)

**Fig. 5:**
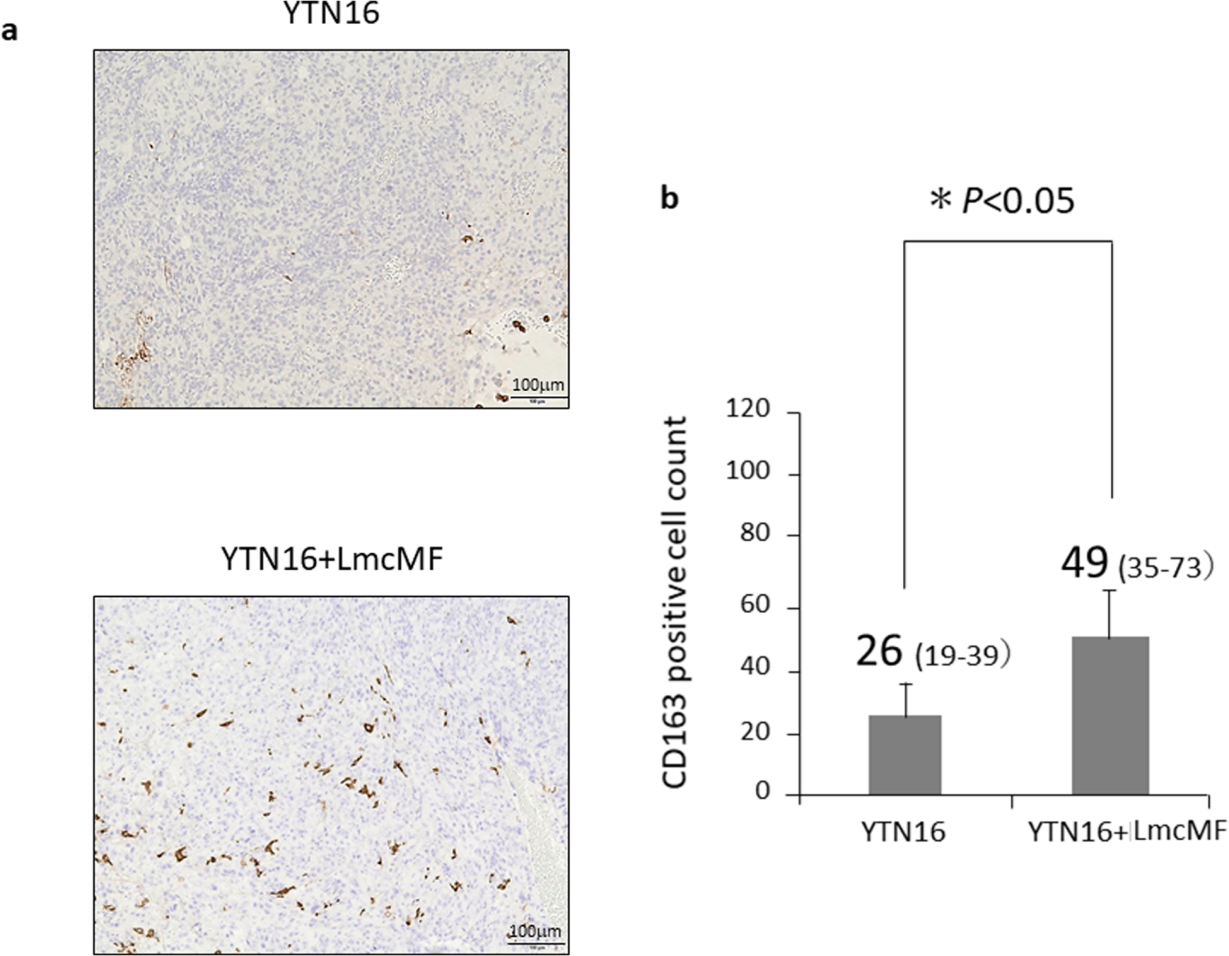
CD163^+^ cell infiltration in each tumors of peritoneal allograft models. **a** Microscopic views of mouse allograft tumors (original magnification × 100). CD163^+^ cells were determined by immunohistochemically. **b** Number of infiltrated CD163^+^ cells were measured and shown as average count of three non-overlapping tumor areas. Results are expressed as the mean ± SD (n = 6)

There was no significant difference in the median CD4^+^ cell counts between tumors from co-inoculation with YTN16 and LmcMF tumor (fibrous tumors) and those from inoculation with YTN16 alone (data not shown). Significantly fewer CD8^+^ cells were found in fibrous tumors than in non-fibrous tumors (median: 64 cells; range 40–68 vs. 9 cells; range 3–15, *P* < 0.05) (Fig. 4, Additional file 3). On the other hand, we found a higher rate of CD163^+^ cell infiltration in fibrous tumors than in non-fibrous tumors (median: 26 cells; range 19–39 vs. 49 cells; range 35–73, *P* < 0.05) (Fig. 5, Additional file 4).

## Discussion

In this clinical analysis, the TME in peritoneal lesions was quite different from the TME in primary lesions. There were significantly fewer CD8^+^ cells corresponding to CTL in peritoneal lesions than in primary lesions. The presence of CD8^+^ cells in the tumor infiltrate prior to the onset of chemotherapy could predict pathological complete response [28, 29]. These results indicate that it is difficult to treat with not only ICIs but also chemotherapy in TME of PM. Stromal fibroblasts so called CAFs suppress CD8^+^ cells function via PDL2 and FAS-ligand, and enhance secretion of interleukin (IL)-6 via crosstalk with cancer cells resulting decreasing accumulation of CD8^+^ cells [30, 31]. Accordingly, it is important to suppress the function of CAFs for recruitment of CD8^+^ cells.

There was no significant difference in the number of CD4^+^ cells between primary and peritoneal lesions. CD4^+^ cells include helper T cells, monocytes, and macrophages, which have various functions in tumor progression. CD4 infiltration status is not statistically associated with prognosis [32], but may affect the efficacy of ICIs.

CD163^+^ cells corresponding to M2 macrophages were found to be significantly higher in PM than in primary lesions. Although macrophages are thought to function as both antitumor agents (M1 macrophages) and protumor agents (M2 macrophages) [33], protumoral tumor-associated macrophages (TAMs) are believed to exhibit characteristics similar to M2 macrophages [34]. Monocytes migrate into peritoneal lesions using the C-C chemokine ligand 2 (CCL2) derived from CAFs and differentiate into the M2 phenotype by macrophage colony-stimulating factor (M-CSF) and IL-6 secreted from CAFs and cancer cells [35]. These M2 macrophages contribute to tumor angiogenesis, immune suppression, and metastasis [36, 37]. The high density of M2 macrophages in PM may contribute to the low efficacies of ICIs treatments for metastatic GC, including PM.

Low-dose paclitaxel changed macrophage phenotype from M2 to M1 through the toll-like receptor (TLR)-4, resulting in tumor growth inhibition [38, 39]. This theory is consistent with taxan-containing therapies showing superior results than other regimens for treating gastric cancer [40, 41]. Half of the patients with unresectable GC have PM at the time of second-line chemotherapy induction [2, 41]. Because ICI was used after treatment failure of taxan-containing therapies, the phenotype of macrophages may have changed from M1 to M2 in the PM TME. Therefore, another strategy for reprogramming macrophages from the M2 to M1 phenotype is needed.

We established a fibrous peritoneal tumor model by co-inoculating the mouse gastric cancer cell line YTN16 and the mouse myofibroblast cell line LmcMF into immune-competent C57BL6/J mice. LmcMF cells played as CAFs by crosstalk with YTN16 resulted in creating much more extracellular matrix represented by collagen fiber than tumors inoculated with YTN16 alone. Tissue fibrosis may interfere with drug delivery and immune cells infiltration due to high intratumor pressure [14]. In the YTN16 and LmcMF co-inoculated tumor model, we observed less infiltration of CD8^+^ cells, but more infiltration of M2 macrophages. In addition, the tumors were hyperprogressive and more invasive compared to YTN16 inoculated tumors. These results indicate that CAFs interfere accumulation of CD8^+^ cells and induce recruitment and M2 polarization of macrophages by secreting various cytokines and/or chemokines. Thus, the importance of CAFs in PM was clarified by demonstrating difference of TME in both mouse model with or without LmcMF cells.

This study has some limitations. First, the number of clinical samples was small, due to difficulty of obtaining non-treated paired specimens from GC patients with PM. Second, TME of biopsy specimens from the surface of the primary tumors may differ from serosal site specimens. Third, TME differences between solid peritoneal tumors were investigated for only one cell line. However, to our knowledge, this is the first report of the establishment of a fibrous peritoneal model in immunocompetent mice.

## Conclusion

In this study, we have clarified the TME of PM in GC and successfully established a mouse model that closely resembles human clinical findings. By using this model, it could be possible to develop new treatment strategies through anti-CAFs therapy.

## Supplementary information

**Additional file 1.** α-SMA stained peritoneal tumor sample in YTN16 plus LmcMF inoculated mouse model. Visualized at 100 magnification, showing stromal fibroblasts as CAFs.

**Additional file 2.** Azan stained peritoneal tumor samples in YTN16 alone and YTN16 plus LmcMF inoculated mouse models. Visualized at 200 magnification, showing stromal fibrosis.

**Additional file 3.** CD8 stained peritoneal tumor samples in YTN16 alone and YTN16 plus LmcMF inoculated mouse models. Visualized at 100 magnification, showing CD8^+^ cells as CTLs.

**Additional file 4.** CD163 stained peritoneal tumor samples in YTN16 alone and YTN16 plus LmcMF inoculated mouse models. Visualized at 200 magnification, showing CD163^+^ cells as M2 macrophages.

## Data Availability

The datasets used and/or analyzed during the current study are available from the corresponding author on reasonable request.

## Abbreviations

PM: Peritoneal metastasis
GC: Gastric cancer
TME: Tumor immune microenvironment
CAFs: Cancer-associated fibroblasts
EMT: Endothelial mesenchymal transduction
ICIs: Immune checkpoint inhibitors
NSCLC: Non-small cell lung carcinoma
PD-1: Programmed death-1
TAMs: Tumor-associated macrophages
MNU: *N*-Methyl-*N*-nitrosourea
DMEM: Dulbecco’s modified Eagle medium
FBS: Fetal bovine serum
H&E: Hematoxylin and eosin
CCL2: C-C chemokine ligand 2
M-CSF: Macrophage colony-stimulating factor
TLR: Toll-like receptor

## References

[1] Bray F, Ferlay J, Soerjomataram I, Siegel RL, Torre LA, Jemal A (2018). Global cancer statistics 2018: GLOBACAN estimates of incidence and mortality worldwide for 36 cancers in 185 countries. CA Cancer J Clin.

[2] Wilke H, Muro K, Van Custsem E, Oh SC, Bodoky G, Shimada Y (2014). Ramucirumab plus paclitaxel versus placebo plus paclitaxel in patients with previously treated advanced gastric or gastro-oesophageal junction adenocarcinoma (RAINBOW): a double blind, randomized phase 3 trial. Lancet Oncol.

[3] Shirao K, Boku N, Yamada Y, Yamaguchi K, Doi T, Goto M (2013). Randomized phase III study of fuluorouracil continuous infusion vs. sequential methotrexate and 5-fluorouracil therapy in far advanced gastric cancer with peritoneal metastasis (JCOG0106). Jpn J Clin Oncol.

[4] Iwase S, Nakajima TE, Nakamura K, Takashima A, Kato K, Hamaguchi T (2012). First-line fluorouracil-based chemotherapy for patients with severe peritoneal disseminated gastric cancer. Gastric Cancer.

[5] Fushida S, Kinoshita Y, Yagi Y, Funaki H, Kinami S, Ninomiya I (2008). Dual-cancer effects of weekly intraperitoneal docetaxel in treatment of advanced gastric cancer patients with peritoneal carcinomatosis: a feasibility and pharmacokinetic study. Oncol Rep.

[6] Ishigami H, Kitayama J, Kaisaki S, Hidemura A, Kato M, Otani K (2010). Phase II study of weekly intravenous and intraperitoneal paclitaxel combined with S-1 for advanced gastric cancer with peritoneal metastasis. Ann Oncol.

[7] Fushida S, Kinoshita J, Kaji M, Hirono Y, Goda F, Yagi Y (2013). Phase I/II study of intraperitoneal docetaxel plus S-1 for the gastric cancer patients with peritoneal carcinomatosis. Cancer Chemother Pharmacol.

[8] Cho H, Ryu MH, Kim KP, Ryoo BY, Park SR, Kim BS (2017). Phase I/II study of a combination of capecitabine, cisplatin, and intraperitoneal docetaxel (XP ID) in advanced gastric cancer patients with peritoneal metastasis. Gastric Cancer.

[9] Yonemura Y, Fujimura T, Nishimura G, Falla R, Sawa T, Katayama K (1996). Effects of intraoperative chemohyperthermia in patients with gastric cancer with peritoneal dissemination. Surgery..

[10] Yang XJ, Huang CQ, Suo T, Mei LJ, Yang GL, Cheng FL (2011). Cytoreductive surgery and hyperthermic intraperitoneal chemotherapy improves survival of patients with peritoneal carcinomatosis from gastric cancer final results of a phase III randomized clinical trial. Ann Surg Oncol.

[11] Sugarbaker PH (1995). Peritonectomy procedures. Ann Surg.

[12] Yonemura Y, Kawamura T, Bandou E, Takahashi S, Sawa T, Matsuki N (2005). Treatment of peritoneal dissemination from gastric cancer by peritonectomy and chemohyperthermic peritoneal perfusion. Br J Surg.

[13] Saito H, Fushida S, Harada S, Miyashita T, Oyama K, Yamaguchi T (2018). Importance of human peritoneal mesothelial cells in the progression, fibrosis, and control of gastric cancer: inhibition of growth and fibrosis by tranilast. Gastric Cancer.

[14] Carr RM, Fernandez-Zapico ME (2016). Pancreatic cancer microenvironment, to target or not to target?. EMBO Mol Med.

[15] Yashiro M, Chung YS, Nishimura S, Inoue T, Sowa M (1996). Fibrosis in the peritoneum induced by scirrhous gastric cancer cells may act as “soil” for peritoneal dissemination. Cancer..

[16] Ma Y, Zhu J, Chen S (2018). Activated gastric cancer-associated fibroblasts contribute to the malignant phenotype and 5-FU resistance via paracrine action in gastric cancer. Cancer Cell Int.

[17] Ziani L, Chouaib S, Thiery J (2018). Alteration of the antitumor immune response by cancer-associated fibroblast. Front Immunol.

[18] Hamid O, Robert C, Daud A, Hodi FS, Hwu WJ, Kefford R (2013). Safety and tumor responses with Iambrozumab (anti-PD-1) in melanoma. N Engl J Med.

[19] Motzer RJ, Escudier B, McDermott DF, George S, Hammers S, Srinivas S (2015). Nivolumab versus everolimus in advanced renal-cell carcinoma. N Engl J Med.

[20] Borghaei H, Paz-Ares L, Horn L, Spigel DR, Steins M, Ready NE (2015). Nivolumab versus docetaxel in advanced nonsquamous non-small-cell lung cancer. N Engl J Med.

[21] Ansell SM, Lesokhin AM, Borrello I, Scott EC, Gutierrez M (2015). PD-1 blockade with nivolumab in relapsed or refractory Hodgkin’s lymphoma. N Engl J Med.

[22] Kang YK, Boku N, Satoh T, Ryu MH, Chao Y, Kato K (2017). Nivolumab in patients with advanced gastric or gastro-oesophageal junction cancer refractory to, or intolerant of, at least two previous chemotherapy regimen (ONO-4538-12, ATTRACTION-2): a randomized, double-blind, placebo-controlled, phase 3 trial. Lancet.

[23] Teng MW, Ngiow SF, Ribas A, Smyth MJ (2015). Classifying cancers based on T-cell infiltration and PD-L1. Cancer Res.

[24] Ichikawa H, Nagahashi M, Shimada Y, Hanyu T, Ishikawa T, Kameyama H (2017). Actionable gene-based classification toward precision medicine in gastric cancer. Genome Med.

[25] Chang YH, Heo YJ, Cho J, Song SY, Lee J, Kim KM (2018). Computational measurement of tumor immune microenvironment in gastric adenocarcinomas. Sci Rep.

[26] Yamamoto M, Nomura S, Hosoi A, Nagaoka K, Iino T, Yasuda T (2018). Established gastric cancer cell lines transplantable into C57BL/6 mice show fibroblast growth factor receptor 4 promotion of tumor growth. Cancer Sci.

[27] Kawasaki H, Ohama T, Hori M, Sato K (2013). Establishment of mouse intestinal myofibroblast cell lines. World Gastroenterol.

[28] Seo AN, Lee HJ, Kim EJ, Kim HJ, Jang MH, Lee HE (2013). Tumour-infiltrating CD8^+^ lymphocytes as an independent predictive factor for pathological complete response to primary systemic therapy in breast cancer. Br J Cancer.

[29] Mahmoud S, Paish E, Powe D, Douglas R, Macmillan R, Grainge M (2011). Tumor-infiltrating CD8^+^ lymphocytes predict clinical outcom in breast cancer. J Clin Oncol.

[30] Lakins MA, Ghorani E, Munir H, Martins CP, Shields JD (2018). Cancer- associated fibroblasts induce antigen-specific deletion of CD8^+^ T cells to protect tumour cells. Nat Commun.

[31] Kato T, Noma K, Ohara T, Kashima H, Katsura Y, Sato H (2018). Cancer-associated fibroblasts affect intratumoral CD8^+^ and FoxP3^+^ T cells via IL6 in the tumor microenvironment. Clin Cancer Res.

[32] Zheng X, Song X, Shao Y, Xu B, Chen L, Zhou Q (2017). Prognostic role of tumor-infiltrating lymphocytes in gastric cancer: a meta-analysis. Oncotarget..

[33] Lewis CE, Pollard JW (2006). Distinct role of macrophages in different tumor microenvironments. Cancer Res.

[34] Yamaguchi T, Fushida S, Yamamoto Y, Tsukada T, Kinoshita J, Oyama K (2016). Tumor-associated macrophages of the M2 phenotype contribute to progression in gastric cancer with peritoneal dissemination. Gastric Cancer.

[35] Komohara Y, Jinushi M, Takeya M (2014). Clinical significance of macrophage heterogeneity in human malignant tumors. Cancer Sci.

[36] Matovani A, Allavena P, Sica A, Balkwill F (2008). Cancer-related inflammation. Nature..

[37] Noy R, Pollard JW (2014). Tumor-associated macrophages: from mechanisms to therapy. Immunity..

[38] Yamaguchi T, Fushida S, Yamamoto Y, Tsukada T, Kinoshita J, Oyama K (2017). Low-dose paclitaxel suppresses the induction of M2 macrophages in gastric cancer. Oncol Rep.

[39] Wanderley CW, Colόn DF, Luiz JPM, Oliveira FF, Vicava PR, Leite CA (2018). Paclitaxel reduces tumor growth by reprogramming tumor-associated macrophages to an M1 profile in a TLR4-dependent manner. Cancer Res.

[40] Ishigami H, Fujiwara Y, Fukushima R, Nashimoto A, Yabusaki H, Imano M (2018). Phase III trial comparing intraperitoneal and intravenous paclitaxel plus S-1 versus cisplatin plus S-1 in patients with gastric cancer with peritoneal matastasis: PHOENIX-GC trial. J Clin Oncol.

[41] Takashima A, Shitara K, Fujitani K, Kodera K, Hara K, Nakayama N (2019). Peritoneal metastasis as a predictive factor for nab-paclitaxel in patients with pretreated advanced gastric cancer: an exploratory analysis of the phase III ABSOLUTE trial. Gastric Cancer.

