# Supplementary figures and images for: Established fibrous peritoneal metastasis in an immunocompetent mouse model similar to clinical immune microenvironment of gastric cancer
Source: BMC Cancer. 2020 Oct 20;20:1014. doi: 10.1186/s12885-020-07477-x (PMC7574408; doi:10.1186/s12885-020-07477-x)

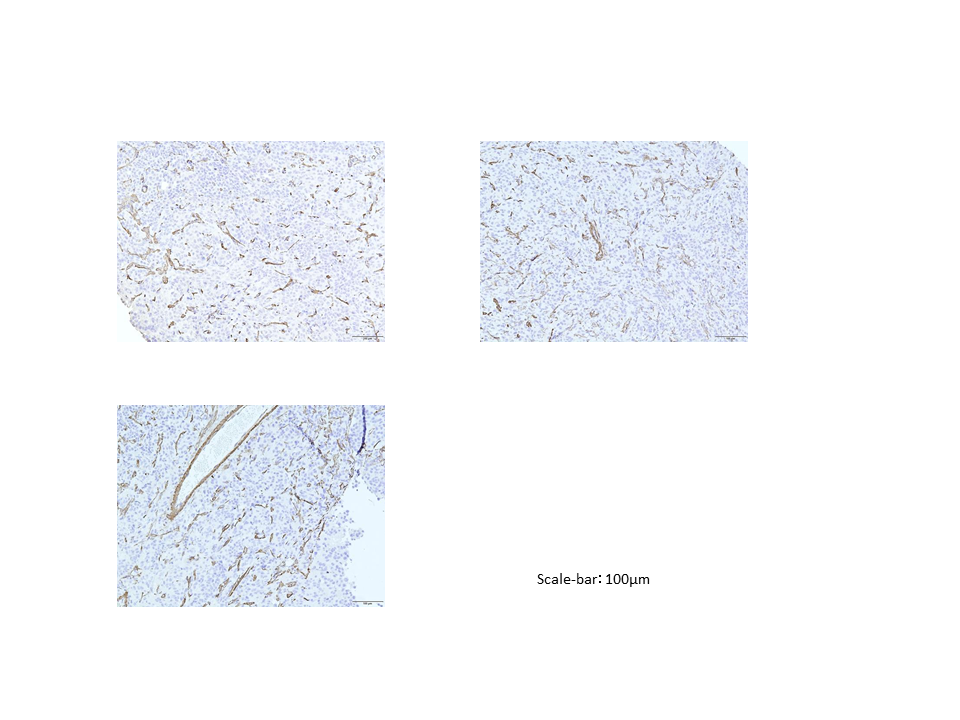

Supplement: Supplementary file 1 — Additional file 1. α-SMA stained peritoneal tumor sample in YTN16 plus LmcMF inoculated mouse model. Visualized at 100 magnification, showing stromal fibroblasts as CAFs. [file 12885_2020_7477_MOESM1_ESM.tif]

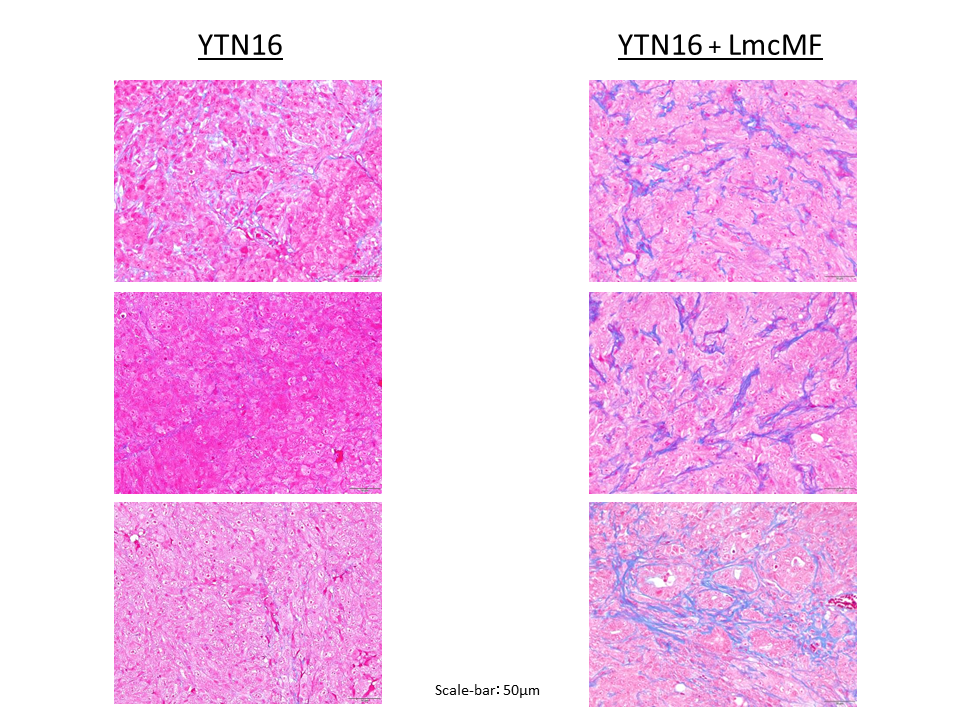

Supplement: Supplementary file 2 — Additional file 2. Azan stained peritoneal tumor samples in YTN16 alone and YTN16 plus LmcMF inoculated mouse models. Visualized at 200 magnification, showing stromal fibrosis. [file 12885_2020_7477_MOESM2_ESM.tif]

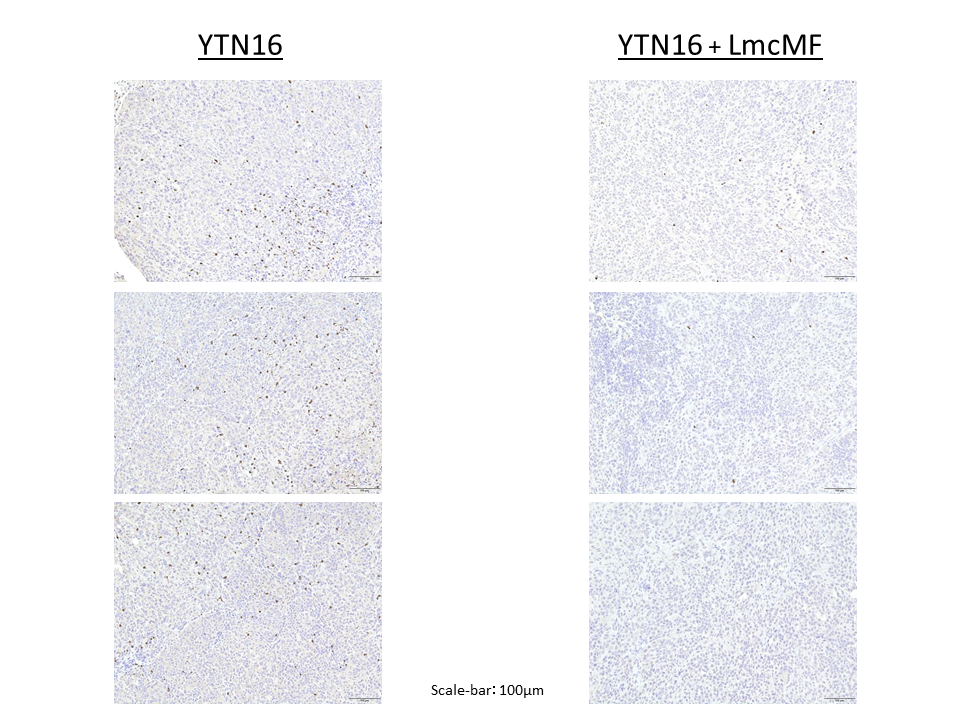

Supplement: Supplementary file 3 — Additional file 3. CD8 stained peritoneal tumor samples in YTN16 alone and YTN16 plus LmcMF inoculated mouse models. Visualized at 100 magnification, showing CD8+ cells as CTLs. [file 12885_2020_7477_MOESM3_ESM.tif]

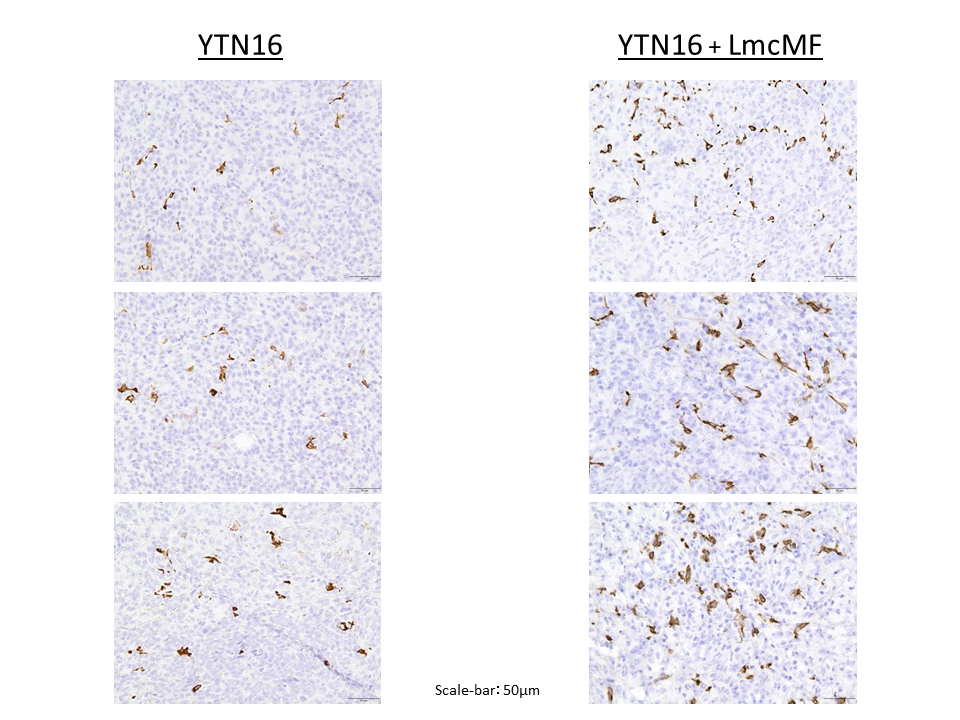

Supplement: Supplementary file 4 — Additional file 4. CD163 stained peritoneal tumor samples in YTN16 alone and YTN16 plus LmcMF inoculated mouse models. Visualized at 200 magnification, showing CD163+ cells as M2 macrophages. [file 12885_2020_7477_MOESM4_ESM.tif]
